# Nox2 contributes to age-related oxidative damage to neurons and the cerebral vasculature

**DOI:** 10.1172/JCI125173

**Published:** 2019-07-22

**Authors:** Lampson M. Fan, Li Geng, Sarah Cahill-Smith, Fangfei Liu, Gillian Douglas, Chris-Anne Mckenzie, Colin Smith, Gavin Brooks, Keith M. Channon, Jian-Mei Li

**Affiliations:** 1 Division of Cardiovascular Medicine, University of Oxford, Oxford, United Kingdom.; 2 School of Biological Sciences, University of Reading, Reading, United Kingdom.; 3 Faculty of Health and Medical Sciences, University of Surrey, Surrey, United Kingdom.; 4 Centre for Clinical Brain Sciences, University of Edinburgh, Edinburgh, United Kingdom.

**Keywords:** Aging, Neuroscience, Apoptosis, Neurodegeneration, endothelial cells

## Abstract

Oxidative stress plays an important role in aging-related neurodegeneration. This study used littermates of WT and Nox2-knockout (Nox2KO) mice plus endothelial cell–specific human Nox2 overexpression–transgenic (HuNox2Tg) mice to investigate Nox2-derived ROS in brain aging. Compared with young WT mice (3–4 months), aging WT mice (20–22 months) had obvious metabolic disorders and loss of locomotor activity. Aging WT brains had high levels of angiotensin II (Ang II) and ROS production; activation of ERK1/2, p53, and γH2AX; and losses of capillaries and neurons. However, these abnormalities were markedly reduced in aging Nox2KO brains. HuNox2Tg brains at middle age (11–12 months) already had high levels of ROS production and activation of stress signaling pathways similar to those found in aging WT brains. The mechanism of Ang II–induced endothelial Nox2 activation in capillary damage was examined using primary brain microvascular endothelial cells. The clinical significance of Nox2-derived ROS in aging-related loss of cerebral capillaries and neurons was investigated using postmortem midbrain tissues of young (25–38 years) and elderly (61–85 years) adults. In conclusion, Nox2 activation is an important mechanism in aging-related cerebral capillary rarefaction and reduced brain function, with the possibility of a key role for endothelial cells.

## Graphical abstract

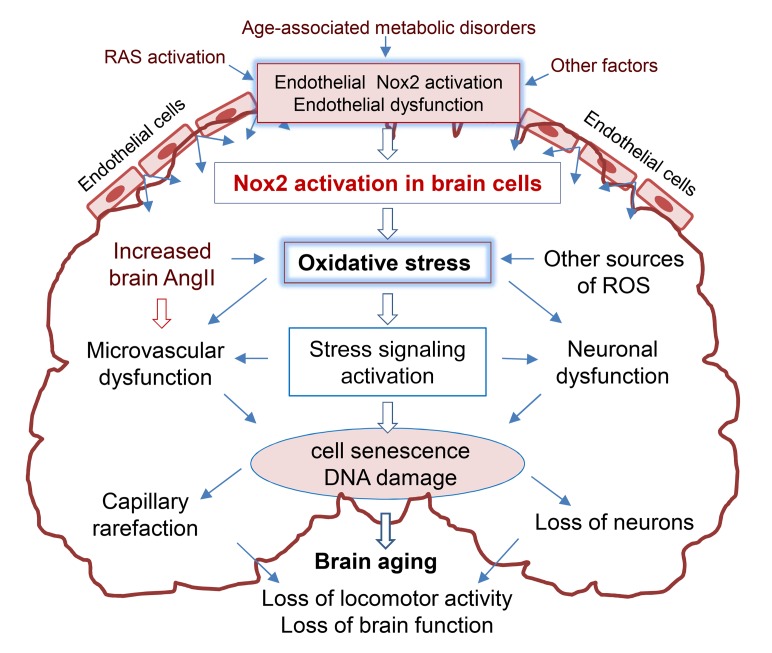

## Introduction

The brain is a highly metabolic organ requiring a consistent supply of oxygen and nutrients for normal function. Ensuring a sufficient oxygen and nutrition supply to the brain is accomplished by highly organized cerebral microvascular networks (1). In humans, the cerebral microvasculature consists of approximately 400 miles of capillaries, and the number of endothelial cells in the brain is similar to that of neurons (2). More importantly, there is growing evidence that impairment of cerebral microvascular perfusion plays a key role in the development of aging-related neurodegenerative diseases (3). The aging process is accompanied by increased oxidative stress in multiple organs and is a major risk factor for cardiovascular diseases (4). Recently, there is growing interest in life stress and local overproduction of angiotensin II (Ang II) in the brain leading to CNS oxidative stress and cerebral vascular damage (5, 6). Abnormal upregulation of brain Ang II activity has also been observed in animal models of aging, menopause, and chronic cerebral hypoperfusion (5–7). Ang II is a potent activator of endothelial Nox2-NADPH oxidase (Nox2).

The NADPH oxidases consists of a cytochrome *b*_558_ (containing a catalytic Nox subunit and a p22^phox^ subunit) and several regulatory subunits, e.g., p40^phox^, p47^phox^, p67^phox^, and rac1 (8). To date, 7 isoforms of Nox (Nox1–5 and Duox 1–2) have been discovered (9, 10). Among them, Nox2 is highly expressed in cells throughout the CNS, including cerebral endothelial cells. Endothelial Nox2 NADPH oxidase has low basal activity under physiological conditions but is activated by stimuli such as Ang II, high glucose, and oxidized LDL. Excessive O_2_^•–^ production by Nox2 causes endothelial dysfunction, which is involved in the development of many vascular diseases, including hypertension, stroke, and cerebral small vessel diseases (11). Endothelial dysfunction characterized by increased ROS production by Nox2 has been recognized as an early feature of aging-related vascular disorders (4, 12, 13). In aging mice, Nox2-derived ROS has been shown to damage endothelium-dependent vessel relaxation (4), and transgenic mice with endothelial cell-specific Nox2 overexpression display high levels of ROS production and ERK1/2 activation in the aorta, with increased susceptibility to Ang II–induced dissection (14, 15). Knockout of Nox2 protects animals from noise- and sleep deprivation–induced endothelial dysfunction and cerebral oxidative stress (16). There is a close relationship between the levels of endothelial oxidative stress and the degree of vascular disorders found in experimental animals and in humans (11, 16, 17). However, in normal aging-associated brain oxidative stress, cerebral microvascular rarefaction, and neuronal degeneration, the mechanism of Nox2 activation in the brain remains unclear.

In this study, we used littermates of age-matched WT and Nox2-knockout (Nox2KO) mice at young (3–4 months; similar to humans at 20–30 years of age) and old age (20–22 months; similar to humans at 70–80 years) to investigate the mechanism of Nox2 activation in oxidative damage of the cerebral microvasculature and locomotor function in aging. Furthermore, we used a mouse model of endothelial cell–specific overexpression of human Nox2–transgenic (HuNox2Tg) mice to examine whether endothelial Nox2 activation plays a role in triggering whole brain oxidative stress and DNA damage in aging. The clinical significance of aging-associated Nox2 activation in brain oxidative stress and cerebral vascular damage was examined using postmortem midbrain tissue samples from young (aged 25–38 years) and elderly (aged 61–85 years) adults. The signaling pathways of Ang II–induced endothelial Nox2 activation and capillary damage were further investigated using primary brain microvascular endothelial cells (BMECs) isolated from middle-aged WT mice in the presence or absence of specific peptide Nox2 inhibitors (Nox2tat). Our study reveals a crucial role for aging-associated brain Nox2 activation in oxidative damage of cerebral vasculature and neurons, with a key role for endothelial cells.

## Results

### Knockout of Nox2 improved global metabolism and preserved locomotor function at old age.

**Figure 1:**
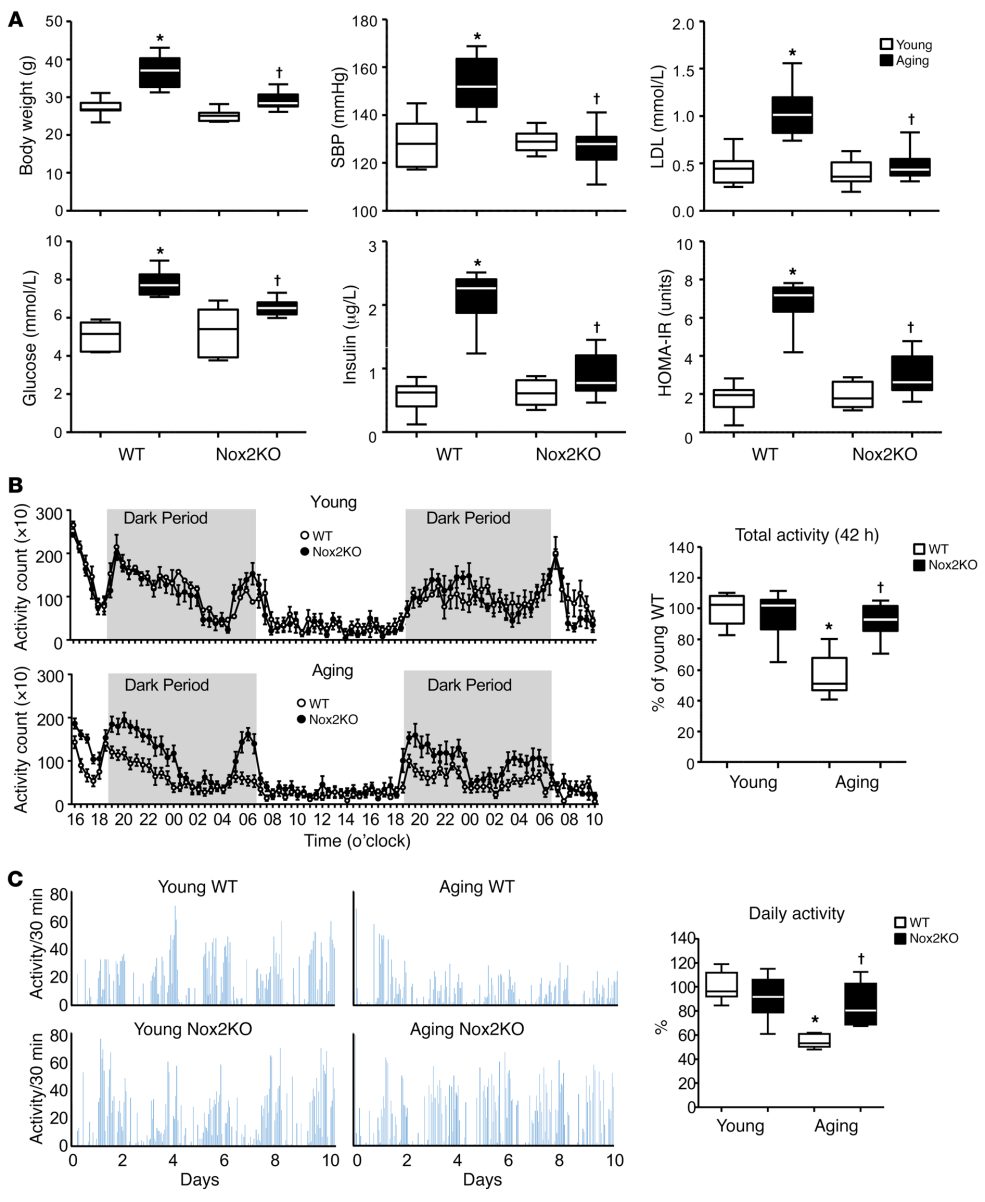
Metabolism and locomotor activities of WT and Nox2KO mice. (**A**) Metabolic measurements. SBP, systolic BP. (**B**) Locomotor horizontal activity measured using motility chambers. Data were collected in 30-minute bins over a 42-hour period. Total activities were calculated and expressed as percent relative to the values of young WT mice (100%). (**C**) Voluntary running wheel activity measured for 10 days. Average daily activity was calculated and expressed as percent relative to the values of young WT mice (100%). *n* = 9 mice. *P < 0.05 for indicated values versus young WT value; ^†^*P* < 0.05 for indicated values versus aging WT values. Statistical analysis was performed using 1-way ANOVA followed by Bonferroni’s post hoc tests.

At young age (3–4 months), there was no significant difference in body weight, BP, fasting serum glucose, insulin, or LDL between WT and Nox2KO mice (Figure 1A). At old age (20–22 months), WT mice had significant increases in body weight, BP, and levels of fasting glucose, insulin, and LDL. Aging WT mice were insulin resistant, as indicated by homeostatic model assessment–insulin resistance (HOMA-IR) scores. However, these age-related metabolic disorders were reduced or absent in aging Nox2KO mice (Figure 1A and Supplemental Figure 1; supplemental material available online with this article; https://doi.org/10.1172/JCI125173DS1).

Locomotor function was examined using motility chambers by measuring the horizontal and vertical activity of mice for a period of 42 hours (the initial 6 hours of establishment period was excluded). A clear diurnal locomotor pattern was shown (Figure 1B). Activity was low in the daytime and increased markedly at night, starting slightly before the expected gradual dimming of the light; and after the high initial activity, the activity of the mice decreased until the end of the light, “resting” phase. At young age, there was no significant difference in locomotor activities between WT and Nox2KO mice; however, at old age, WT mice (but not Nox2KO mice) had notably reduced night horizontal activities (dark period) as compared with young controls (Figure 1B). There was no significant difference in vertical movement activities between aging WT and No2KO mice (Supplemental Figure 2, A–C). There was no significant difference in brain weights between WT and Nox2KO mice for either the young or aging group (Supplemental Figure 2D). Voluntary running wheel activity was examined during a period of 10 days (Figure 1C). Once again, there was no significant difference between WT and Nox2KO mice at young age. However, there was a significant reduction in running wheel activity for aging WT (but not Nox2KO) mice in comparison to their respective young controls.

### Nox2 activation and oxidative damage of cerebral microvasculature and neurons in aging.

**Figure 2:**
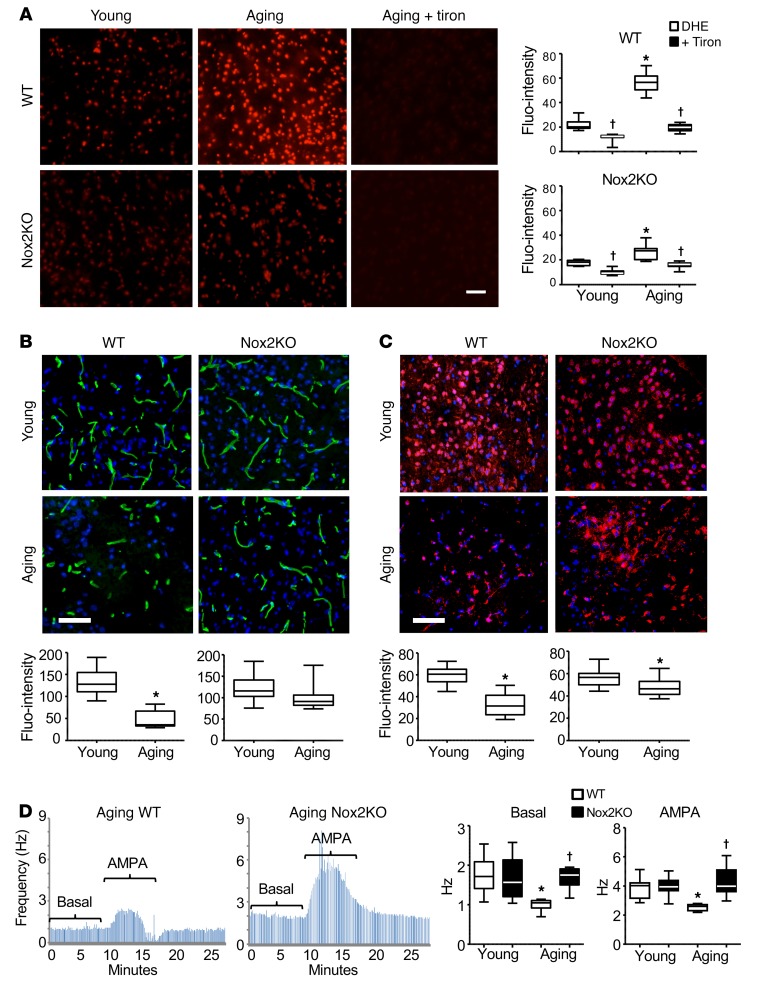
Aging-related changes in WT and Nox2KO mouse midbrains. (**A**) ROS production by mouse midbrain sections detected by DHE fluorescence. Tiron was used to confirm the detection of O_2_^•–^. *P < 0.05 for indicated values versus young DHE values; ^†^*P* < 0.05 for indicated values versus DHE values in the same age group. Fluo-intensity, fluorescence intensity. (**B**) Cerebral microvascular density. Capillaries were labeled with LE-lectin (FITC, green) and quantified. (**C**) Neuronal density. Neurons were labeled with NeuN (Cy3, red) and quantified. Nuclei were labeled with DAPI (blue in **B** and **C**). *n* = 6 mice/per group. Scale bars: 100 μm. (**D**) Dopaminergic neuron firing frequency. Left panels: Representative recording traces of aging brains. Right panels: Statistical analysis. *n* = 14 cells from 6 mice. *P < 0.05 for indicated values versus young values in the same genetic group; ^†^*P* < 0.05 for indicated values versus aging WT values. Statistical analysis was performed using 1-way ANOVA followed by Bonferroni’s post hoc tests.

In order to establish the role of Nox2-derived ROS in aging-associated decline of motor performance shown in Figure 1, B and C, we used sections from the midbrain region (containing the hippocampus and ventral tegmental area [VTA]) to examine in situ ROS production by dihydroethidium (DHE) fluorescence (Figure 2A). At young age, Nox2KO brains had slightly lower ROS production as compared with age-matched WT controls (Figure 2A). In comparison to young WT controls, aging WT brains displayed a remarkable increase (~2.5-fold) in ROS production, and this could be reduced to the levels in young controls by addition of Tiron, an O_2_^•–^ scavenger. The levels of ROS production in aging Nox2KO brains were also increased in comparison to young Nox2KO brains, albeit to a considerably lesser extent than in aging WT brains (Figure 2A, right panels).

We then examined cerebral capillary density (Figure 2B) labeled with *Lycopersicon esculentum* lectin (LE-lectin, specific for *N*-acetyl-d-glucosamine and *N*-acetyl-polylactosamine oligomers), which has been reported to be an effective and versatile endothelial marker of vessels in the CNS (18). For determination of neuronal density (Figure 2C), sections of the same brain regions were labeled with the neuron marker NeuN. We found that increased ROS production by aging WT brains was accompanied by a significant reduction in cerebral capillary density (Figure 2B) and neurons (Figure 2C) in comparison to their respective young controls. However, capillary density and neuronal density were well preserved in aging Nox2KO brain as compared with young Nox2KO controls. Neuronal function was examined by measuring neuron firing frequency in the VTA with or without AMPA (2-amino-3-[3-hydroxy-5-methyl-isoxazol-4-yl]propanoic acid), a specific agonist of the AMPA receptor responsible for fast synaptic transmission in the CNS (19). A representative real-time recording of Nox2KO young brain neuron firing is shown in Supplemental Figure 3. There was no significant difference in either basal (without AMPA) or AMPA-stimulated neuron firing frequencies between young WT and Nox2KO mice (Figure 2D). However, at old age, the neuron firing frequency (both basal and AMPA-stimulated) was remarkably reduced in aging WT brains but well preserved in aging Nox2KO mice (Figure 2D).

### Nox2 subunit expression and activation of stress signaling pathways in aging brains.

**Figure 3:**
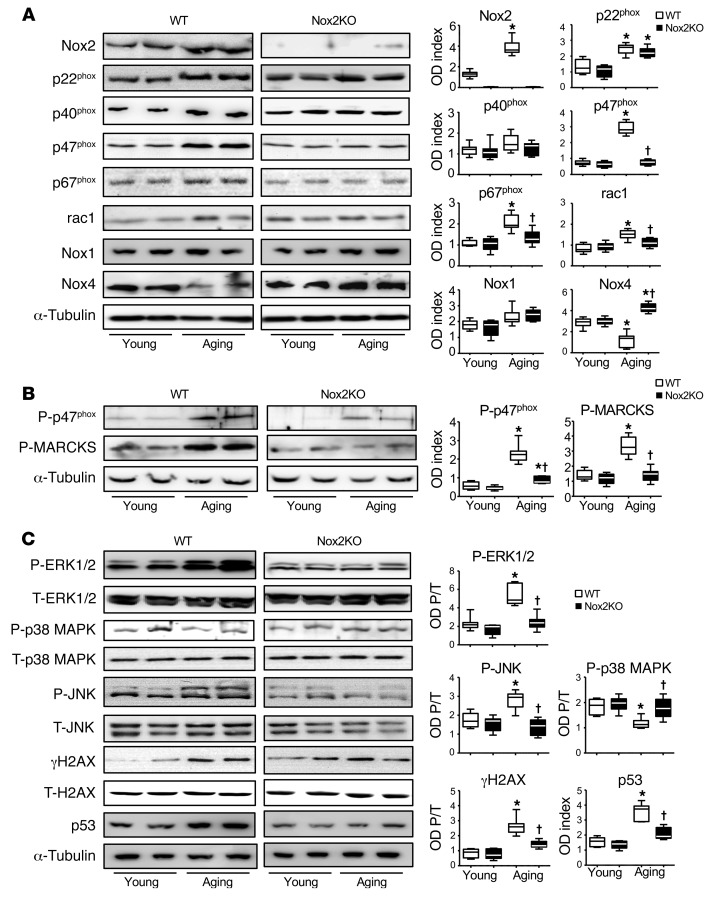
Nox2 subunit expression and activation of stress signaling pathways in mouse midbrain tissues detected by Western blot. (**A**) Nox subunit expression. (**B**) Phosphorylation of p47^phox^ and MARCKS. ODs of protein bands were quantified and normalized to α-tubulin detected in the same sample. (**C**) MAPK activation and DNA damage marker expression. The phospho-bands (P) were quantified and normalized to the total bands (T) of the same protein detected in the same samples, are expressed as OD P/T. The p53 bands were quantified and normalized to α-tubulin detected in the same sample. *n* = 6 mice/group. *P < 0.05 for indicated values versus young values in the same genetic group; ^†^*P* < 0.05 for indicated values versus aging WT values. Statistical analysis was performed using 1-way ANOVA followed by Bonferroni’s post hoc tests.

Levels of Nox2 subunit expression in the midbrain tissue homogenates were examined by Western blotting (Figure 3A). Compared with young WT young brains, there were significant increases in the protein expression of Nox2 and its regulatory subunits, i.e., p22^phox^, p47^phox^, p67^phox^, and rac1; and these were accompanied by a significant decrease in Nox4 expression in aging WT brains. However, in the absence of Nox2, there was no significant difference in the levels of expressions of p47^phox^, p67^phox^, and rac1 between young and aging Nox2KO brains. In addition, aging Nox2KO brains had a significant increase in Nox4 and p22^phox^ expression as compared with their young controls (Figure 3A). There was no significant difference in the levels of Nox1 and p40^phox^ expression between WT and Nox2KO brains for both the young and aging groups. Phosphorylation of p47^phox^ (a key regulatory subunit of Nox2) by PKC is a crucial step in Nox2 activation. Myristoylated alanine-rich protein kinase C substrate (MARCKS) phosphorylation is a marker of PKC activation in the nervous system (20). We found significant increases in the levels of p47^phox^ and MARCKS phosphorylation in aging WT (but not Nox2KO) brains (Figure 3B).

MAPK activation is a crucial pathway for Nox2 signaling. Therefore, we examined ERK1/2, p38 MAPK, and JNK phosphorylation in midbrain tissues. Total levels of the same protein detected in the same samples were used as loading controls (Figure 3C). Compared with those in young WT brains, levels of ERK1/2 and JNK phosphorylation were increased, whereas levels of p38 MAPK phosphorylation were decreased in aging WT brains. Along with the activation of ERK1/2 and JNK, there were significant increases in markers of DNA damage, i.e., increased phosphorylation of the histone variant H2AX at Ser139 to form γH2AX and increased p53 expression in aging WT brains. In contrast, there was no significant difference in MAPK phosphorylation between young and aging Nox2KO brains, and aging Nox2KO brains had less DNA damage as compared with aging WT brains (Figure 3C).

**Figure 4:**
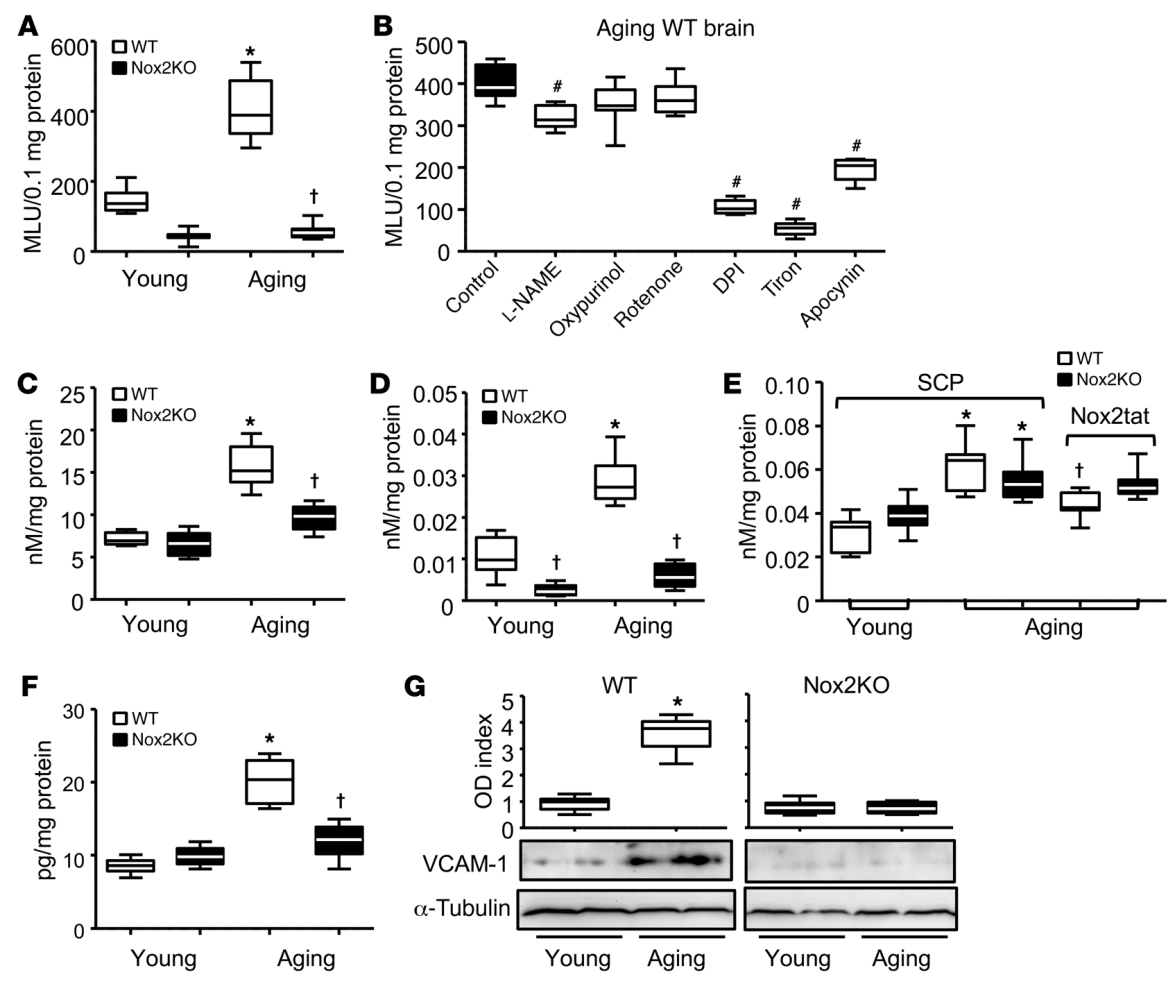
Age-associated increases in ROS production and in levels of Ang II and VCAM-1 expression in midbrain tissues of WT and Nox2KO mice. (**A**) ROS production detected by lucigenin chemiluminescence. (**B**) Inhibitor assay. The effects of different enzyme inhibitors on ROS production by aging WT midbrain tissues detected by lucigenin chemiluminescence. ^#^*P* < 0.05 for indicated values versus aging WT control values without inhibitor. MLU, mean light units. (**C**) Brain tissue lipid peroxidation detected by MDA assay. (**D**) Brain tissue O_2_^•–^ production detected by SOD-inhibitable cytochrome *c* reduction assay. (**E**) Brain tissue H_2_O_2_ production detected by catalase-inhibitable Amplex red assay. Nox2tat was used to inhibit Nox2. (**F**) Brain tissue Ang II levels detected by ELISA. (**G**) Vascular inflammation maker (VCAM-1) expression detected by Western blot analysis. ODs of protein bands were quantified and normalized to α-tubulin detected in the same sample. *n* = 6 mice/group. *P < 0.05 for indicated values versus young values in the same genetic group; ^†^*P* < 0.05 for indicated values versus WT values in the same age group (**A**, **C**, **D**, and **F**) or for indicated values versus values without inhibitor in the same age and genetic group (**E**). Statistical analysis was performed using 1-way ANOVA followed by Bonferroni’s post hoc tests.

Along with Nox2 activation, there were significant (2-fold) increases in NADPH-dependent O_2_^•–^ production by aging WT (but not Nox2KO) brains in comparison to young WT controls as examined by lucigenin chemiluminescence (Figure 4A). Increased O_2_^•–^ production by the aging WT brains could be significantly reduced by adding Tiron, diphenyleneiodonium (DPI; a flavoprotein inhibitor), apocynin (a Nox2 inhibitor), or l-NAME (an eNOS inhibitor), but not rotenone (mitochondrial complex I enzyme inhibitor) or oxypurinol (xanthine oxidase inhibitor) (Figure 4B). Increased ROS production by aging WT brain tissues was further confirmed by increased lipid peroxidation detected by MDA assay (Figure 4C) and SOD-inhibitable cytochrome *c* reduction assay (Figure 4D). Although levels of H_2_O_2_ production detected by Amplex red assay were increased for both aging WT and aging Nox2KO mice (Figure 4E), in the presence of Nox2tat (10 μm/L), the levels of H_2_O_2_ produced by aging WT brain were notably inhibited. Along with the increased ROS production in aging WT brains, there were increases in the levels of brain tissue Ang II (Figure 4F) and the expression of a vascular inflammation marker (VCAM-1) (Figure 4G). Together, our data strongly suggest a crucial role of Nox2-derived O_2_^•–^ in oxidative damage of the cerebral vasculature and neurons in the midbrain region of aging WT mice.

### Experiments using postmortem human midbrain tissues.

**Figure 5:**
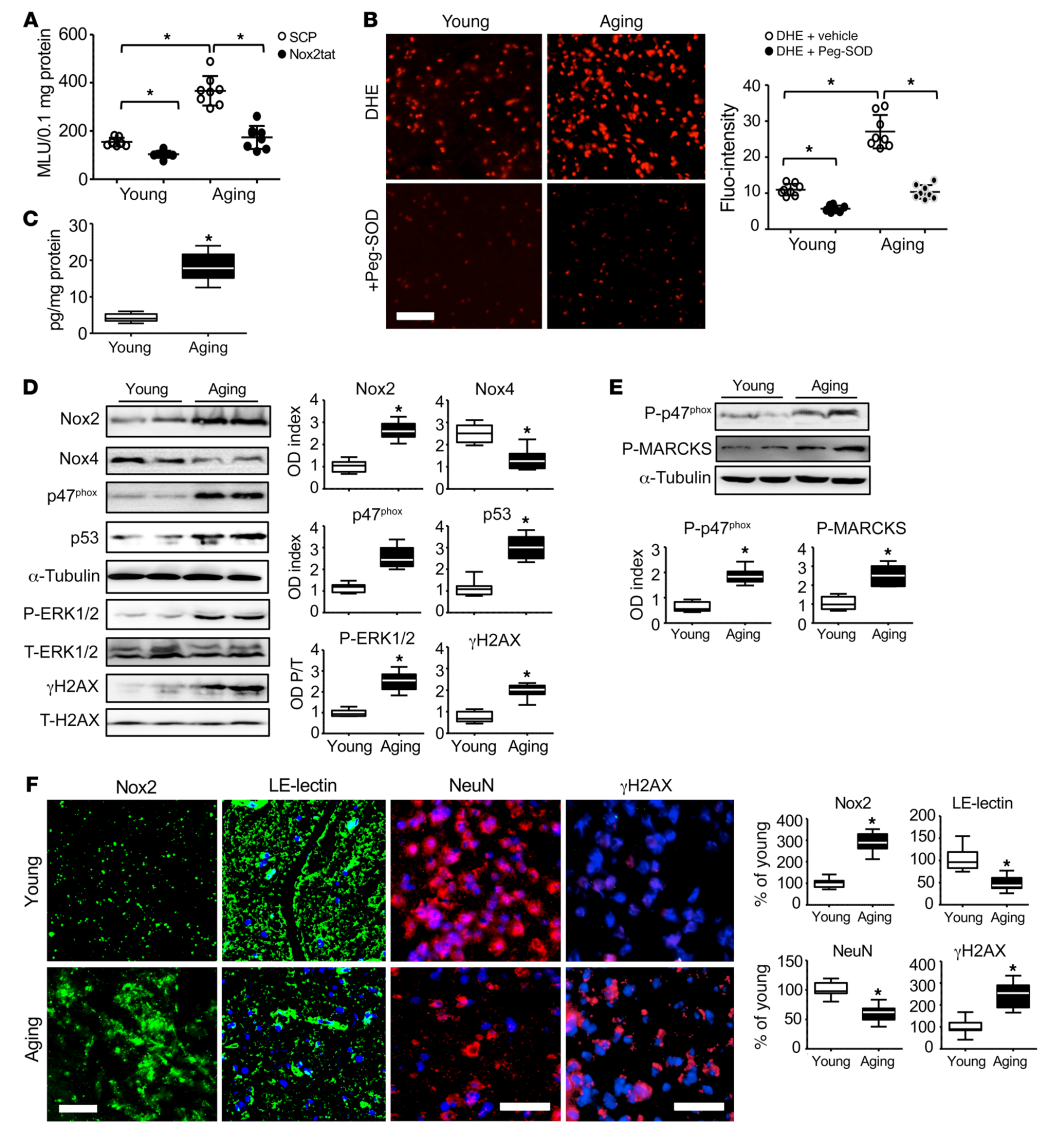
Nox2 expression, activation of stress signaling pathways, and oxidative damage in postmortem human midbrain tissues. (**A**) ROS production detected by lucigenin chemiluminescence in the presence of an SCP or Nox2tat. (**B**) ROS production by midbrain sections detected by DHE fluorescence with or without Peg-SOD. *P < 0.05 between indicated group values. (**C**) Brain tissue Ang II levels detected by ELISA. (**D**) Nox subunit and p53 expression and phosphorylation of ERK1/2 and H2AX (to form γH2AX) detected by Western blot analysis. (**E**) p47^phox^ and MARCKS phosphorylation detected by Western blot analysis. ODs of protein bands were quantified and normalized to α-tubulin detected in the same samples. The phospho-ERK1/2 and γH2AX bands were normalized to the total protein bands detected in the same samples, expressed as OD P/T. (**F**) Immunofluorescence on midbrain sections. Nox2 was labeled with FITC; cerebral vessels were labeled with LE-lectin (FITC); neurons were labeled with NeuN (Cy3, red); and γH2AX was labeled by Cy3 (red). Nuclei were labeled with DAPI (blue) to visualize cells. Scale bars: 100 μm. *n* = 7–8 individual brains and 2 sections/per brain. Fluorescence intensities were quantified and expressed as percent relative to young (100%). *P < 0.05 for indicated values versus young values (**C**–**F**). Statistical analysis was performed using 1-way ANOVA followed by Bonferroni’s post hoc tests.

The clinical significance of Nox2 activation in aging-related oxidative damage of human brain microvascular vessels and neurons was examined using postmortem human midbrain tissues (including the hippocampus and VTA) of young (25–38 years old), middle-aged (45–56 years old), and elderly (61–85 years old) adults without diagnosed neurodegenerative diseases, obtained from the UK Medical Research Council (MRC) tissue bank. The demographics of human brain tissues are shown in Supplemental Table 2. It was noted that 5 of 6 middle-aged and 5 of 8 elderly adults had aging-related metabolic and cardiovascular disorders. There was a significant increase compared with young controls in brain O_2_^•–^ production starting at middle age (Supplemental Figure 4) and a further increase (~2-fold) at old age, which could be reduced to young control levels by addition of Nox2tat (Figure 5A). Increased aging brain O_2_^•–^ production was inhibited by SOD, DPI, Tiron, or l-NAME (Supplemental Figure 4). Increased ROS production by human aging brains was further confirmed by in situ DHE fluorescence on brain sections in the presence or absence of polyethylene glycol–SOD (Peg-SOD) (Figure 5B) and MDA assay (Supplemental Figure 5). Along with increased ROS production, there were significant increases in brain tissue Ang II levels (Figure 5C); upregulation of Nox2, downregulation of Nox4, and activation of stress signaling pathways (Figure 5D); and increased phosphorylation of p47^phox^ (a key step in Nox2 activation) and MARCKS (a marker of PKC activation) (Figure 5E). Immunofluorescence further demonstrated that increased Nox2 expression in the aging midbrain region was accompanied by significant losses in cerebral capillary density (labeled with LE-lectin) and neurons (labeled with NeuN) and increased DNA damage (labeled with γH2AX) in comparison to young controls (Figure 5F). Our human data provided strong support for the discoveries in animals (Figures 1–4) that Nox2 activation played a key role in oxidative damage of cerebral microvasculature and neurons in aging.

### Experiments using midbrain tissues of endothelial cell–specific expression of human Nox2-transgenic mice at young and middle ages.

Nox2 is highly expressed in endothelial cells, and the number of endothelial cells in the brain is similar to that of neurons (2). We have found increased brain Ang II levels in aging WT mice (Figure 4F) and humans (Figure 5C), and Ang II is a potent activator of endothelial Nox2. In order to investigate whether endothelial Nox2 activation could trigger whole brain oxidative stress and cerebral vasculature rarefaction in aging, we used midbrain tissues from middle-aged (11–12 months) HuNox2Tg mice. Endothelium-specific overexpression of the human Nox2 transgene was confirmed by real-time PCR (Supplemental Figure 6). Endothelial cell–dependent ROS production was confirmed by endothelium denudation of HuNox2Tg aortas stimulated with Ang II (Supplemental Figure 7). Cerebral endothelial (labeled with LE-lectin) expression of human Nox2 mRNA in HuNox2Tg brains was confirmed by in situ hybridization using RNAscope technology (Supplemental Figure 8).

**Figure 6:**
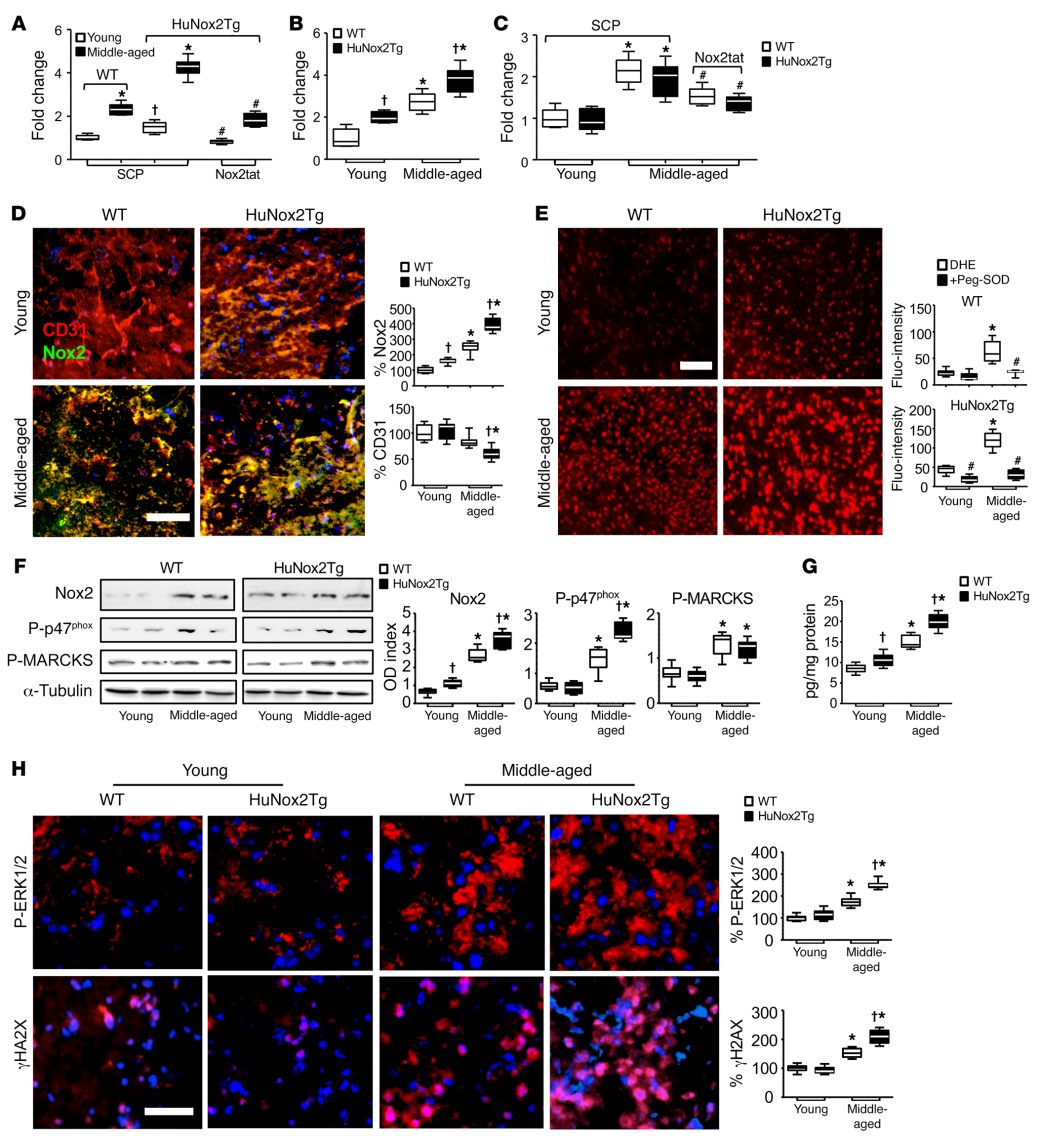
Nox2 activity and activation of stress signaling pathways in WT and HuNox2Tg mouse midbrain tissues. (**A**) ROS production detected by lucigenin chemiluminescence in the presence of SCP or Nox2tat. (**B**) O_2_^•–^ production as detected by SOD-inhibitable cytochrome *c* reduction assay. (**C**) H_2_O_2_ production detected by catalase-inhibitable Amplex red assay. (**D**) Endothelial Nox2 expression. Endothelial cells were labeled with CD31 (red), and Nox2 was labeled with FITC. Yellow represents endothelial Nox2 expression. (**E**) ROS production as detected by DHE fluorescence on midbrain sections. (**F**) p47^phox^ and MARCKS phosphorylation. ODs of protein bands were quantified and normalized to α-tubulin detected in the same sample. (**G**) Brain tissue Ang II levels as detected by ELISA. (**H**) Immunofluorescence detection of phospho-ERK1/2 (red, top row) and γH2AX (red, bottom row). Nuclei were labeled with DAPI (blue). Pink indicates the nuclear location of γH2AX. Scale bars: 100 μm. *n* = 6 mouse brains and 3 sections/per brain. *P < 0.05 for indicated values versus young values in the same genetic group; ^†^*P* < 0.05 for indicated values versus WT values of the same age group; ^#^*P* < 0.05 for indicated values versus values without inhibitor in the same age and genetic group. Statistical analysis was performed using 1-way ANOVA followed by Bonferroni’s post hoc tests.

In comparison to age-matched WT controls, young HuNox2Tg brains had slightly higher levels of ROS production (Figure 6A), and this was greatly increased at middle age, with levels similar to those detected in aging WT brains (Figure 4, A, C, and D), as examined by 5 complementary techniques, i.e., lucigenin chemiluminescence (Figure 6A), SOD-inhibitable cytochrome *c* reduction assay (Figure 6B), Amplex red assay (Figure 6C), in situ DHE fluorescence (Figure 6E), and MDA assay (Supplemental Figure 9). Increased O_2_^•–^ production by middle-aged HuNox2Tg brains could be markedly inhibited by Nox2tat (Figure 6, A and C), which further confirmed a role for Nox2 activation. Increased Nox2 protein expression (green) in cerebral endothelium (labeled with CD31, red) of HuNox2Tg midbrain sections was further confirmed by superposed immunofluorescence (Figure 6D, yellow). There was a significant decrease in CD31 immunofluorescence in middle-aged huNox2Tg brain sections, suggesting endothelial damage (Figure 6D). In comparison to age-matched WT controls, middle-aged HuNox2Tg brains showed significant increases in brain Nox2 expression and p47^phox^ phosphorylation as detected by Western blot analysis (Figure 6F) and increased brain Ang II levels detected by ELISA (Figure 6G). Redox-sensitive ERK1/2 phosphorylation (Figure 6H top row) and γH2AX formation (a marker of DNA damage) were increased in nuclei of middle-aged HuNox2Tg brains (Figure 6F, bottom row, pink) in comparison to age-matched WT controls.

### Experiments using BMECs isolated from middle-aged WT mice.

**Figure 7:**
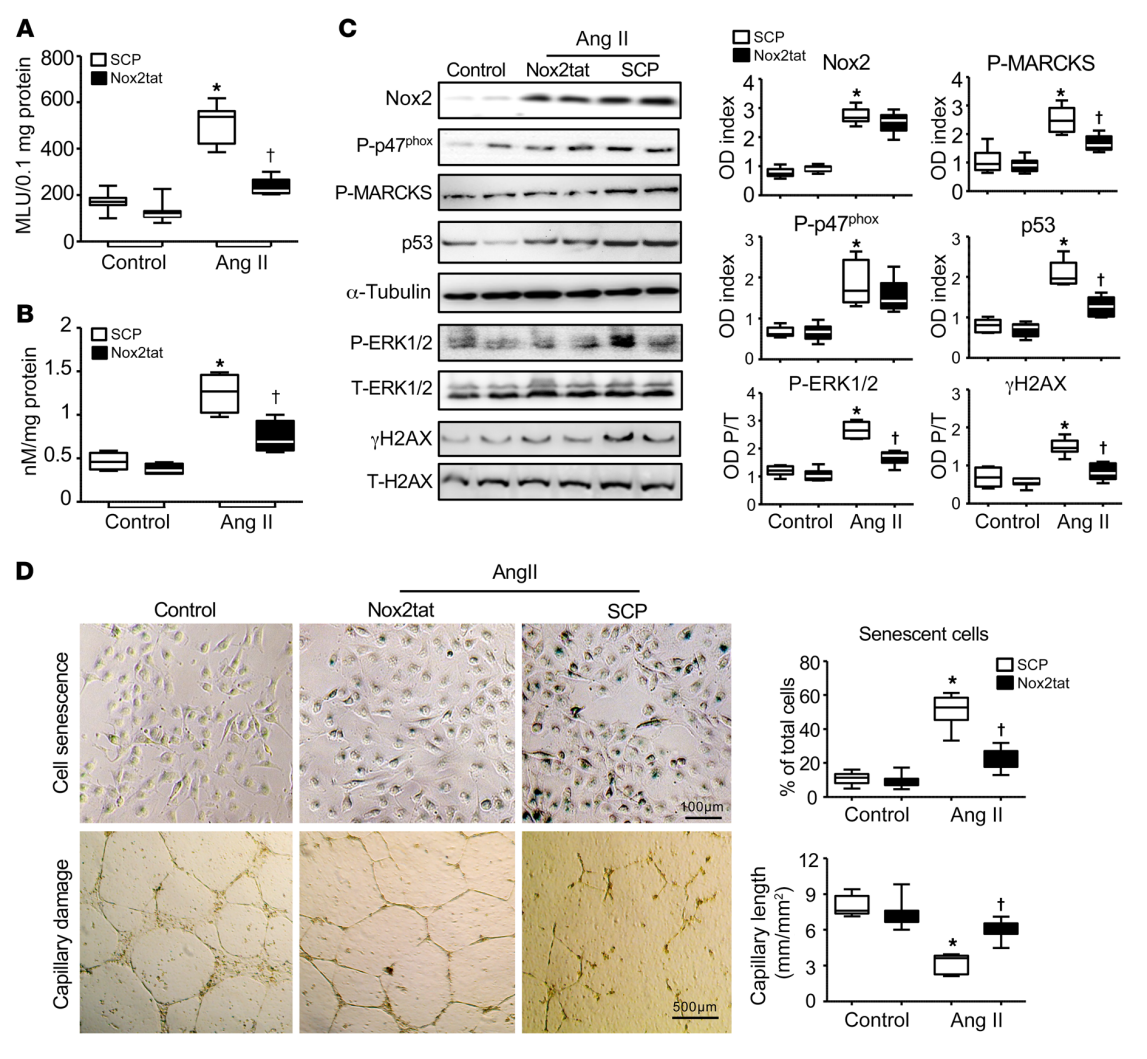
Ang II–induced Nox2 activation in mediating cell senescence and capillary damage of BMECs. (**A**) BMEC O_2_^•–^ production detected by lucigenin chemiluminescence in the presence of SCP or Nox2tat. (**B**) Lipid peroxidation in BMEC homogenates as detected by MDA assay. (**C**) Nox2 expression and activation of stress signaling pathways as detected by Western blot analysis. ODs of protein bands were quantified and normalized to α-tubulin detected in the same samples. The phospho-ERK1/2 and γH2AX bands were normalized to the total protein bands detected in the same samples, expressed as OD P/T. (**D**) Cell senescence and capillary damage. Top row: BMEC senescence as detected by SAβG activity assay (blue). Scale bar: 100 μm. Bottom row: Ang II–induced capillary damage on Matrigels. Scale bar: 500 μm. *P < 0.05 for indicated values versus control values in the same treatment group; ^†^*P* < 0.05 for indicated values versus Ang II SCP values. *n* = 4 separate BMEC isolations/group. Three mice were used for each BMEC isolation. Statistical analysis was performed using 1-way ANOVA followed by Bonferroni’s post hoc tests.

Our data from both animals and humans had revealed an age-related increase in brain Ang II levels together with Nox2 activation and cerebral microvascular damage in aging brains. Ang II is well known to cause endothelial dysfunction through the activation of Nox2. In order to further explore the mechanism of Ang II in cerebral endothelial dysfunction and brain capillary rarefaction in aging, we isolated BMECs from middle-aged WT mice and challenged cells with Ang II (100 μM, for 24 hours) in the presence of scrambled control peptides (SCPs) or Nox2tat (Figure 7). Compared with vehicle control, Ang II remarkably increased the levels of BMEC ROS production as detected by lucigenin chemiluminescence (Figure 7A) and lipid peroxidation as detected by MDA assay (Figure 7B). Increased ROS production was accompanied by increased Nox2 expression; phosphorylation of p47^phox^, MARCKS (marker of PKC activation), ERK1/2; and increased γH2AX and p53 expression as detected by Western blot analysis (Figure 7C). However, all these Ang II–induced oxidative responses were inhibited in the presence of Nox2tat. We then examined Ang II–induced endothelial senescence and capillary damage. In comparison to control, Ang II increased the numbers of senescent cells detected by senescence-associated β-gal (SAβG) activity (blue, Figure 7D, top row) and capillary damage on Matrigel (Figure 7D, bottom row). However, this Ang II–induced damage to BMECs was inhibited in the presence of Nox2tat.

## Discussion

The brain is a highly metabolic organ that requires tight regulation of its blood perfusion and redox homeostasis for normal function. Cumulative brain oxidative stress causes damage to the cerebral microvasculature and neurons and is an important pathophysiological manifestation in human aging (12, 21). However, the enzymatic source and mechanisms (or factors) of excess production of ROS in the aging brain remain unclear. This study using age-matched littermates of young (3–4 months) and aging (20–22 months) WT and Nox2KO mice; brain tissues from endothelial cell–specific huNox2Tg mice; and postmortem human midbrain tissues at ages of 25–38 years versus 61–85 years demonstrated that (i) brain oxidative stress attributable to the activation of Nox2-NADPH oxidase plays a key role in aging-related cerebral capillary rarefaction, loss of neurons, and locomotor dysfunction; and (ii) increased endothelial cell ROS production due to endothelial Nox2 overexpression can trigger brain oxidative stress and DNA damage as early as in middle age.

Aging is associated with metabolic disorders and decreased control in locomotor function and increases the risk of developing neurodegenerative diseases. NADPH oxidase is highly expressed in brain cells including cerebral endothelial cells and is involved in the regulation of brain function by generating ROS (22–24). Long-term physical inactivity has been shown to activate endothelial NADPH oxidase, resulting in cerebral vascular dysfunction in mice (25). On the other hand, routine locomotor activity reduces brain oxidative stress (26). Adding to this knowledge about redox regulation of brain function, the current study showed that the normal aging process is associated with a massive increase in brain Nox2-dependent ROS production, together with a reduction in locomotor function, including voluntary wheel running activity in aging WT but not Nox2KO mice.

Voluntary wheel running is not only a general spontaneous aerobic exercise for mice but also reflects several underlying behavioral processes, including motivation (27). Dopaminergic neurons in the VTA are important for cognition, motivation, and control of locomotor activity (28). Reduced dopaminergic neuron firing capability is involved in aging-related behavior disorders, including impaired motor activity and loss of motivation of voluntary exercise (19, 29). Inhibition of Nox2 enzyme or knockout of p47^phox^ (a key regulatory subunit of NADPH oxidase) protected dopaminergic neurons from degeneration and improved behavior and locomotor activity (23, 30). In accordance with these previous studies, we found an aging-related loss of neurons and damage to the dopaminergic neuronal electrophysiological properties of aging WT mice. In stark contrast, knocking out Nox2 protected neurons from oxidative damage and preserved dopaminergic neuron firing function in aging. Our data strongly suggest a crucial role for Nox2-derived ROS in aging-related loss of brain function. The diurnal rhythm of locomotor activity was present in all groups of mice. However, aging WT mice (but not Nox2KO mice) lost the secondary peak in activity just before the beginning of the light “resting” phase. Although our data suggested that Nox2-derived ROS might be involved in the regulation of the circadian rhythm, further detailed investigation is required to understand the underlying mechanisms.

Cerebral endothelial cells have unique properties in terms of blood-brain barrier function, interactions with other types of neurovascular cells, and regulation of local blood flow through release of signaling molecules (31). Increased cerebral endothelial cell ROS production damages brain vascular homeostasis, contributing critically to aging-related neurodegeneration (32). By labeling cerebral microvessels using LE-lectin, we showed in this study an inverse relationship between increased brain Nox2 activity and reduced cerebral capillary density in the midbrain regions of aging WT mice as well as in elderly humans. Although dysfunctional eNOS was also involved in aging brain ROS production (inhibited by l-NAME), by using Nox2KO mice, we showed that Nox2-derived ROS contributed to a far greater extent to oxidative damage of cerebral vessels and neurons. Nox4 had been reported to be implicated in animal models of cerebral ischemia and reperfusion injury after stroke (33). In contrast to the acute injury model of stroke, aging is a slow process, and there was a compensatory increase in Nox4 expression in the aging Nox2KO brain. Further detailed study is needed to fully address the role of Nox4 in brain aging. The MAPK family is an important signaling pathway of Nox2-derived ROS involved in many brain physiological and pathological processes, including maintenance of neuronal viability, vascular alteration, and apoptosis (34–37). Increased ERK1/2 expression has been found in age-related neurodegenerative abnormalities (36). In support of these previous studies, we found Nox2-dependent ERK1/2 activation together with a significant increase in brain cell DNA damage, i.e., increased γH2AX and p53, in the midbrains of aging WT mice and elderly humans.

An important discovery in this study is that increased endothelial cell ROS production due to Nox2 activation can trigger midbrain oxidative stress and promote brain cell DNA damage and premature aging, which has important implications for our current understanding and treatment of vascular neurodegenerative disorders. Increased endothelial ROS production is a key early characteristic component of endothelial dysfunction and is predictive of many clinical vascular events (15, 38). By using brain tissues of endothelial cell–specific HuNox2Tg mice (14), we demonstrated a substantial increase in brain ROS production as early as in middle age, with ROS levels equivalent to those detected in aging WT brains. Furthermore, there was significant ERK1/2 activation and DNA damage indicated by γH2AX formation in middle-aged HuNox2Tg brains in comparison to age-matched WT controls. The clinical significance of Nox2-dependent ROS production in human brain aging was further demonstrated using postmortem midbrain tissues of young (25–38 years old) and elderly (61–85 years old) adults who had died of non-neurodegenerative diseases. We demonstrated a clear age-related significant increase in Nox2-dependent O_2_^•–^ production in these elderly human brains, which could be markedly inhibited by a specific Nox2 inhibitor (Nox2tat) or by Peg-SOD. Once again, we showed that accompanying the upregulation of Nox2 were activation of stress signaling pathways, capillary rarefaction, and loss of neuronal density in aging human midbrain tissues.

All components of the renin-angiotensin system are present within the CNS, and local production of brain Ang II is now well accepted to contribute to the development of hypertension at old age (39, 40). Ang II has been established as a potent activator of endothelial Nox2. In this study, we found a close relationship between brain tissue levels of Ang II and Nox2-derived ROS for both mice and humans. Using primary BMECs isolated from middle-aged WT mice, we further showed that in response to Ang II stimulation, BMECs showed a significant, 3-fold increase in ROS production, and this was accompanied by significant Nox2 activation, cell senescence, and capillary damage on Matrigels. However, the Ang II–induced endothelial damage was reduced in the presence of Nox2tat.

In conclusion, by using several complementary approaches (including in vivo whole animal pathophysiology and in vitro BMEC culture; comparing WT mice with Nox2KO and HuNox2TG mice; and comparing human postmortem brain tissues of young and elderly adults), our study provides insight into the mechanisms by which Nox2-derived ROS regulates brain aging. Increased endothelial Nox2–derived ROS production possibly plays a key role in triggering brain oxidative stress, DNA damage, and brain aging. These findings have important implications for our understanding and management of vascular neurodegenerative disorders.

## Methods

### Reagents.

Details of primary and secondary antibodies used in this study are shown in Supplemental Table 1. DHE was from Invitrogen. The SCP and Nox2tat ([H]-RKKRRQRRRCSTRVRRQL-[NH2]) were provided by PeptideSynthetics (Peptide Protein Research Ltd.). All other reagents and chemicals were from Sigma-Aldrich unless otherwise stated

### Animals.

Nox2KO mice on a C57BL/6J background were originally obtained from the Jackson Laboratory. Nox2KO mice lack phagocyte superoxide production and manifest an increased susceptibility to infection. Littermates of WT and Nox2KO mice were bred at the University of Surrey from heterozygotes and genotyped. Animals were housed under standard conditions with a 12-hour-light/12-hour dark cycle, and food and water were available ad libitum. Male mice were randomly grouped (*n* = 10–12/per group) and used at young age (3–4 months), middle age (11–12 months), and old age (20–22 months) for the experiments. Endothelial cell–specific expression (under the murine Tie2 promoter) of human Nox2-transgenic (HuNox2Tg) mice was generated in the laboratory of Keith Channon (Oxford University) as described previously (14). Brain tissues obtained from littermates of age-matched WT and HuNox2Tg mice at young (3–4 months) and middle age (11–12 months) (*n* = 6/per group) were used for the experiments.

### Human midbrain tissue collection.

Postmortem human midbrain tissues from males and females who died of non-neurodegenerative diseases were obtained from the UK MRC Edinburgh Brain and Tissue Bank headed by Colin Smith (University of Edinburgh). Samples from midbrain regions (including the hippocampus and VTA) without gross pathological abnormalities were collected and grouped randomly according to age: young adult (25–38 years, *n* = 7); middle-aged (45–56 years, *n* = 6); and elderly (61–85 years, *n* = 8). Demographics of human brain tissues are shown in Supplemental Table 2.

### Metabolic assessments.

Metabolic assessments were performed as described previously (4, 41). BP was measured by a computer-controlled noninvasive tail-cuff BP system (Kent Scientific Corp.) on conscious mice (after 1 week of training) at 10 am, and measurements were recorded by the CODA program. Serum glucose was measured at 9 am after 8 hours of fasting using a blood glucose meter (Accu-Chek). Fasting plasma insulin was measured using a mouse insulin ELISA kit (Mercodia). Fasting serum cholesterol and HDL cholesterol were measured by enzymatic colorimetric assays using an ILab 650 Chemistry System (Instrumentation Laboratory). LDL cholesterol was calculated as the difference between total and HDL cholesterol concentrations based on the Friedewald equation (42).

### Locomotor activity measurement.

Mice were housed individually in motility chambers (Linton Instrumentation) at 18–23°C and 45%–55% humidity with a 12-hour-light/12 hour-dark cycle, and food and water were available ad libitum. The horizontal and vertical activities of the mice were measured using 2 sets of 16 photocells located at right angles to each other, projecting horizontal infrared beams 2.5 cm apart and 1 and 6 cm above the cage floor. The lower beams at 1 cm measured horizontal activity, and the upper beams at 6 cm measured vertical, rearing activity. Activities were measured as sequential infrared beam breaks and recorded in 30-minute bins for 42 hours on AmonLite activity monitoring software (MJS Technology Ltd.). For the voluntary wheel running activities, mice were housed individually with open access to a running wheel mounted in the cage (ClockLab, ActiMetrics). Running wheel activities in 2-minute bins for a period of 10 days were recorded using passive infrared (PIR) detection (RISCO Ltd.) and analyzed using ClockLab software.

### Dopaminergic neuron firing by extracellular electrophysiology.

The extracellular electrophysiology experiments were performed as described previously (43). Coronal slices (300 μm) containing the substantia nigra and VTA were prepared using a vibratome (VT1200, Leica) in ice-cold oxygenated electrophysiology buffer containing 123 mM NaCl, 22 mM NaHCO_3_, 1.25 mM NaH_2_PO_4_, 3.75 mM KCl, 10 mM d-glucose, 2.5 mM CaCl_2_, and 1.2 mM MgSO_4_. Single-cell extracellular recordings using glass microelectrodes filled with buffer (impedance of 3–6 MΩ) were captured using an Axopatch 1D (Axon Instruments) in I = 0 mode with a low cutoff frequency of 2 kHz and then further amplified by 100 times in the AC mode using a Neurolog system (Digidata) without any further filtering. Signals were then digitized using a CED 1401 plus (Cambridge) and captured using Spike2 software. Dopaminergic neurons in the VTA were distinguished by a firing frequency of 0.5–4 Hz, an action potential waveform of approximately 2.5–3 ms in duration, and inhibition of firing in response to 50 μM dopamine perfusion. A baseline firing frequency was recorded for 5–10 minutes. AMPA (0.5 μM) was then perfused and recorded for approximately 6 minutes. The AMPA was then washed out, and firing frequency was left to return to its basal rate. After 10–15 minutes, 50 μM dopamine was perfused, which blunted dopaminergic neuron firing. The dopamine was washed out to allow firing to return to normal, which ensured that the blunting of the signal was due to the dopamine perfusion. The results were analyzed manually using Spike2 software.

### Mouse BMEC isolation.

BMECs were isolated from the brains of middle-aged (11–12 months) WT mice according to a protocol published previously, with some modifications (44). Briefly, brain tissues (*n* = 3) were cut into pieces (2 mm^3^) and digested in a 50-mL Falcon tube with 6 mL digestion buffer containing Liberase Blendzyme (0.1 U/mL) and DNAse (1 μg/mL) for 8 minutes at 37°C while shearing through the bore of a 10-mL pipette every 3 minutes. At the end of digestion, the supernatant containing cells and microvessels was transferred into a new tube containing 1 mL ice-cold FCS to stop the digestion. The digestion was repeated 4 times until no visible tissue pieces were left in the tube. Tubes were spun at 120 *g* for 10 minutes. The supernatant containing myelin was discarded, and the pellets were titrated through a 21-gauge needle 3 times. After washing, BMECs were cultured in flasks coated with collagen type I. BMECs were cultured in the DMEM/Nutrient Mixture F-12 medium supplemented with 10% FBS, EC growth supplement (30 μg/mL), EGF (10 ng/mL), VEGF (0.5 ng/mL), ascorbic acid (1 μg/mL), hydrocortisone (1 μg/mL), l-glutamine (2 mM), penicillin (50 U/mL), and streptomycin (50 μg/mL). BMECs were cultured for 3–4 weeks before being used for experiments.

### In situ detection of SAβG activity.

Activity of SAβG (a cell senescence marker) in cultured BMECs was performed as described previously (45). Briefly, cells were cultured on chamber slides and treated according to the experimental design. After fixation with 1% ice-cold paraformaldehyde and washing in PBS, cells were incubated with freshly prepared staining buffer containing 40 mM citric acid/sodium (pH 6.0), 0.15 M NaCl, 2 M MgCl_2_, 5 mM potassium ferrocyanide, and 1 mg/mL X-gal (5-bromo-4-chloro-3-indolyl β-d-galactopyranoside). SAβG-positive cells (blue) were examined microscopically and counted.

### Capillary tube formation on Matrigels.

Study of capillary tube formation on Matrigels was performed according to a previously published method (46). Matrigel was purchased from Sigma-Aldrich (E6909, ECM Gel) and prepared according to the manufacturer’s instructions. Soluble Matrigel (100 μL) was then added using precooled tips into a 48-well plate to cover the wells evenly. BMECs (5 × 10^3^/well) were then seeded into the wells and cultured in complete growth medium overnight. The following morning, cells were treated according to the experimental conditions for 48 hours. The rings per well was counted, and cells were photographed under phase-contrast microscopy.

### ROS measurement.

ROS production by brain (or cerebral endothelial cell) homogenates was measured using 3 complementary techniques (47): (i) lucigenin (5 μM) chemiluminescence in tissue homogenates detected using a 96-well microplate luminometer (Molecular Devices); (ii) detection of in situ DHE (2 μM) fluorescence on tissue sections in the presence or absence of Tiron (10 mM, a nonenzymatic O_2_^•–^ scavenger) to confirm the detection of O_2_^•–^ using a Zeiss fluorescence microscope and quantified from at least 5 random fields/section with 3 sections/sample and 6 animals/group; and (iii) SOD (200 U/mL)–inhibitable cytochrome *c* reduction assay. The enzymatic sources of ROS production were further examined using inhibitors targeting NOS (l-NAME, 100 μM), the mitochondrial complex I enzymes (rotenone, 50 μM), xanthine oxidase (oxypurinol, 250 μM), flavoproteins (DPI, 20 μM), or Nox2 (peptide inhibitor Nox2tat, 10 μM), or an SCP (10 μM). SOD (200 U/mL) was used to confirm the detection of O_2_^•–^.

### Immunoblotting.

Immunoblotting was performed exactly as described previously (41). Midbrain tissue homogenates were used for immunoblotting. For BMEC experiments, cell homogenates were used for immunoblotting. The images were captured digitally using a BioSpectrum AC imaging system (UVP), and the optical densities of the protein bands were normalized to the loading control bands and quantified.

### Immunofluorescence microscopy.

Immunofluorescence microscopy experiments were performed exactly as described previously (4, 41). Primary antibodies were used at 1:250 dilution. BSA (2%) was used in place of primary antibodies as a negative control. Biotin-conjugated anti-rabbit or anti-goat IgG (1:1000 dilution) were used as secondary antibody. Specific binding of antibodies was detected by ExtrAvidin-FITC or streptavidin-Cy3. Images were acquired with either an Olympus BX61 fluorescence microscope system or a Zeiss fluorescence microscope system (Axio Scope.A1). Fluorescence intensities were quantified digitally.

### Detection of brain tissue Ang II and MDA assay.

Ang II levels and lipid peroxidation were detected in the midbrain tissue homogenates using an Ang II EIA kit and an MDA assay kit, respectively (Sigma-Aldrich) according to the manufacturer’s instructions.

### Statistics.

Statistical analysis was performed using 1-way ANOVA followed by Bonferroni’s post hoc tests, except where otherwise specified in the figure legends. Animal metabolic data were obtained from at least 9 mice/group. For the human study, 7–8 individual brain samples were used in each group. Data are presented as box (first and third quartiles) and whiskers (maximum and minimum). The line in the box represents the median. For data not presented as box and whiskers, mean ± SD was used. *P* values less than 0.05 were considered statistically significant.

### Study approval.

The animal studies were performed under the Home Office animal work project licence (PPL 70/6729 and PPL 70/7638) in accordance with protocols approved by both the Ethics Committee of the University of Surrey and the Home Office under the Animals (Scientific Procedures) Act 1986, United Kingdom. The postmortem human brain tissues were obtained from the UK MRC Edinburgh Brain and Tissue Bank. This study received ethical approval from the UK National Health Service Research Ethical Committee (East of Scotland Ethics Service) and the Ethics Committee of the University of Edinburgh.

## Author contributions

LMF, LG, SCS, and FL contributed experimental data acquisition and analysis. LMF drafted the manuscript. GD, KMC, CAM, and CS collected and provided research samples. GB helped in providing research reagents and facilities and in manuscript preparation. JML designed and directed the study and critically reviewed the manuscript.

## Supplementary Material

Supplemental data

## Version 1 07/22/2019

Electronic publication

## Version 2 08/01/2019

Print issue publication
