# Supplementary material for: Nox2 contributes to age-related oxidative damage to neurons and the cerebral vasculature
Source: J Clin Invest. 2019 Jul 22;129(8):3374–86. doi: 10.1172/JCI125173 (PMC6668817; doi:10.1172/JCI125173)
Supplement: Supplemental data [file jci-129-125173-s290.pdf]

Supplemental Figures I-IX

Supplemental Figure I: Age-related increases in body weight and blood pressure.

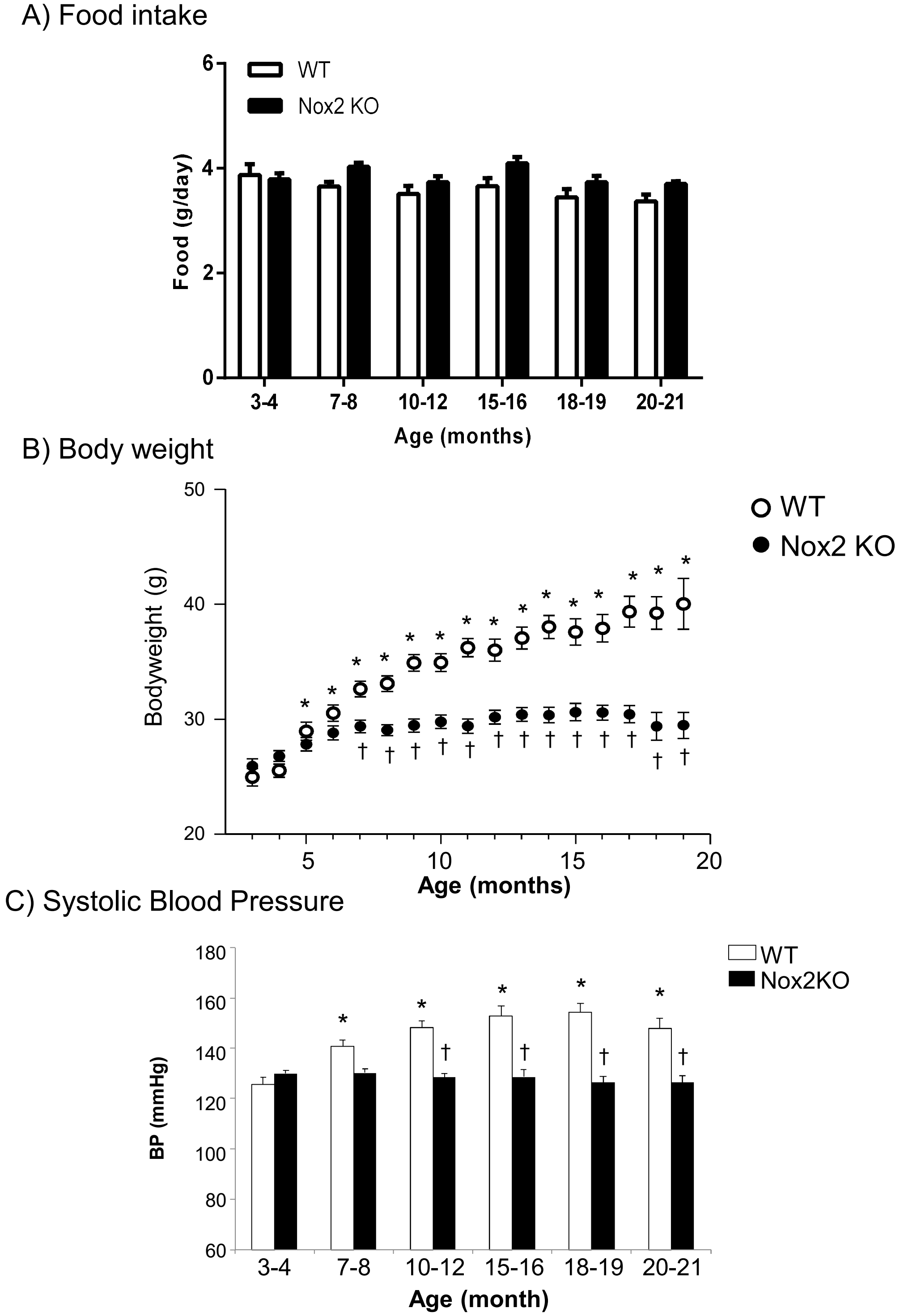

Figure I. (A) Daily food intake was measured in 3 month intervals. The mean of 3 individual 24 hour food intakes was used for each mouse. B) Bodyweight was measured every month. C) Blood pressure was measured in 3 month intervals. Data expressed as mean ± SEM, n=9 mice per group. Comparisons made by two-way ANOVA with bonferroni post-hoc test. *p<0.05 for indicated values versus values of 3-4m WT mice; † p<0.05 for indicated Nox2KO values versus values of age-matched WT mice

Supplemental Figure II: Vertical movement activity and brain weight of WT and Nox2KO mice

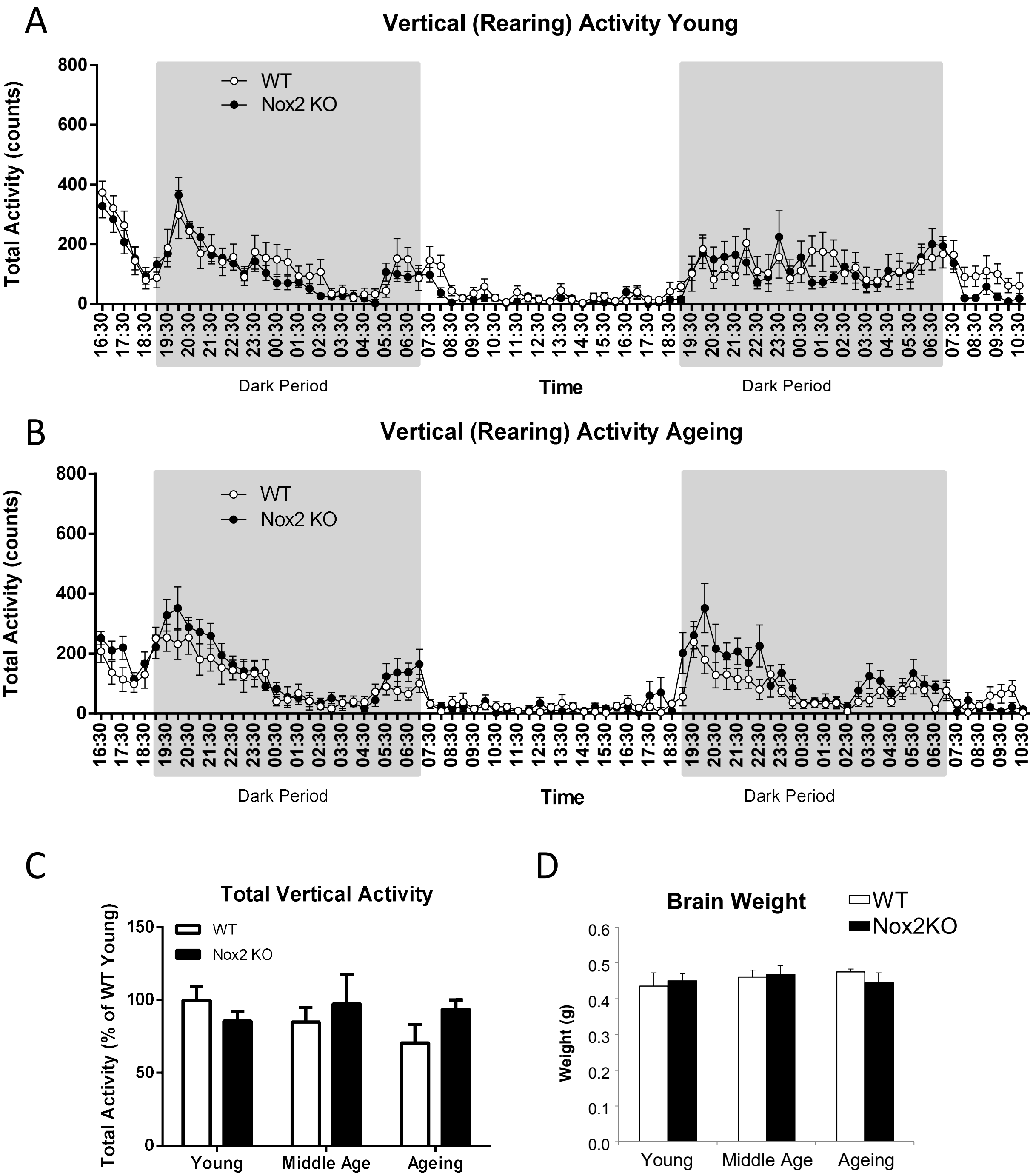

Figure II. Representative graphs of vertical (rearing) locomotor activities of WT and Nox2KO mice. (A) Young mice; (B) Ageing mice. The data was collected in 30-min bins over a 42 hour period. (C) Total vertical activity (42h). Data were expressed as % of WT young mice (100%). (D) Brain weight . n=8-10 mice per group.

Supplemental Figure III: Representive recording of a Nox2KO young DA neuron firing recording

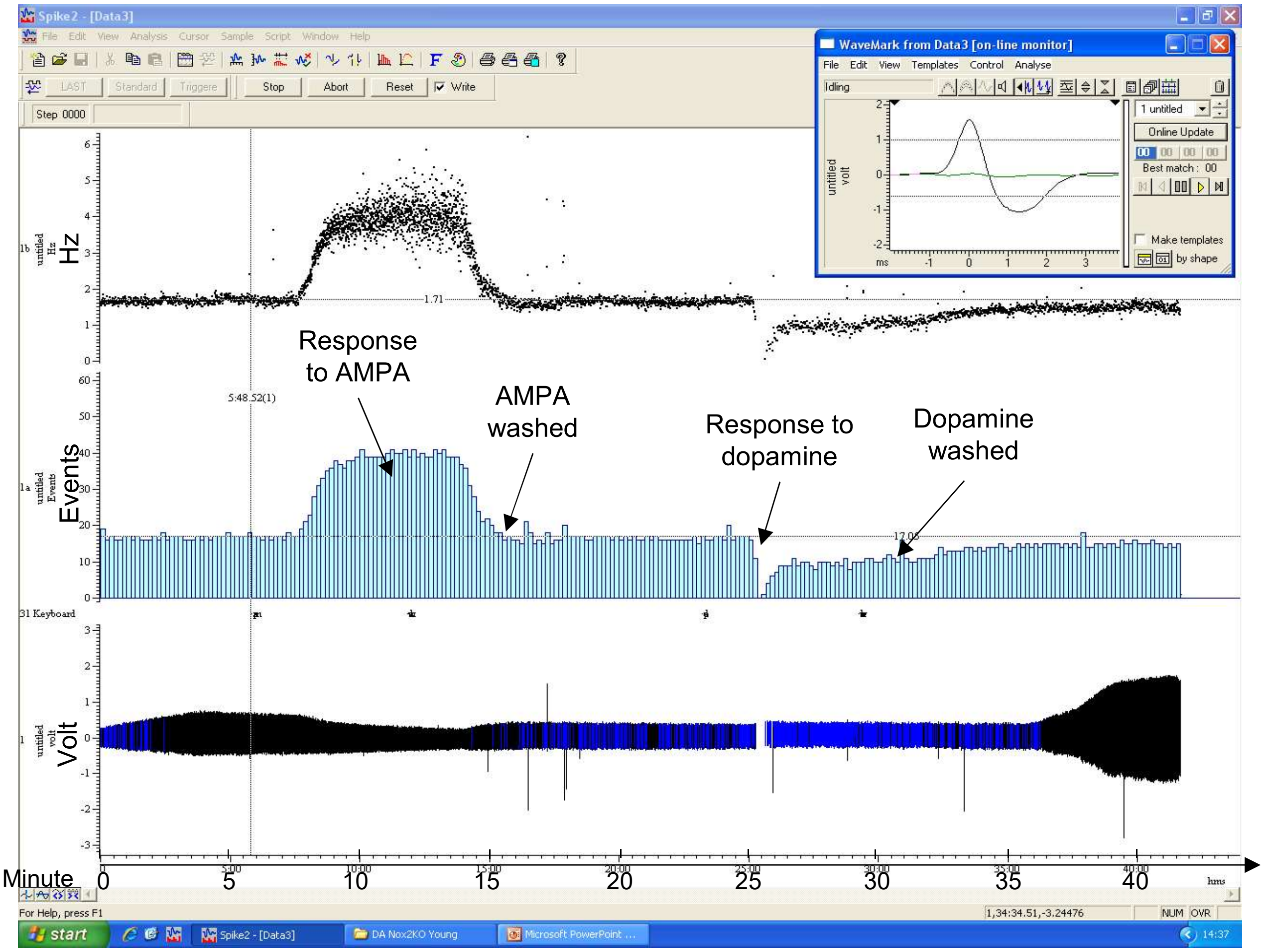

Figure III. Representative real-time recording (screen shot) of a young Nox2KO dopaminergic neuron firing. A baseline firing frequency was recorded for 5-10 minutes. AMPA was then perfused and recorded for ~6 min. The AMPA was then washed out and firing frequency was left to return to its basal rate. After 10-15 minutes 50 µM dopamine was perfused, which blunted dopaminergic neuron firing. The dopamine was washed out to allow firing to return to normal that ensured the blunting of the signal was due to the dopamine perfusion. The results were analyzed manually on the Spike 2 Software.

Supplemental Figure IV: Effects of enzyme inhibitor on human brain ROS production detected by lucigenin-chemiluminescence

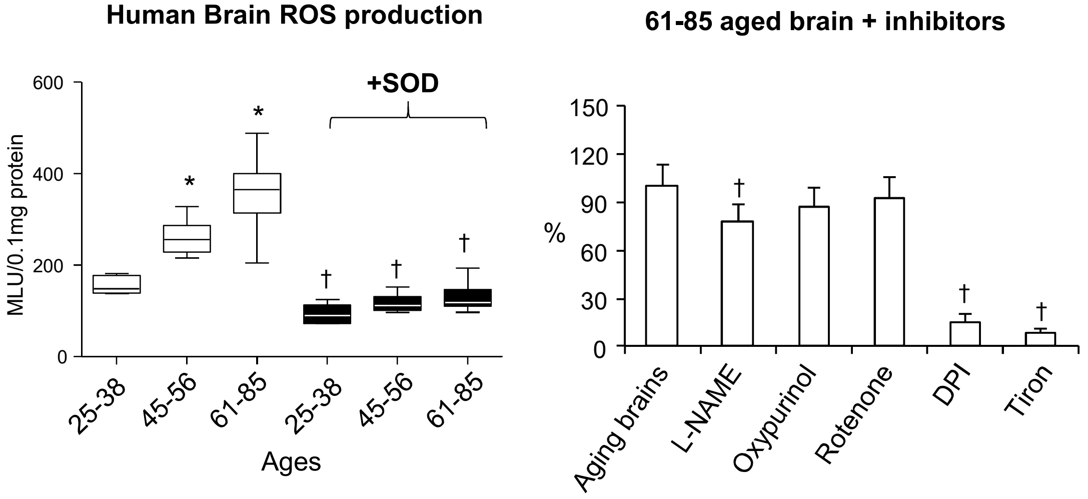

Figure IV: Left panel: ROS production by human brain homogenates detected by lucigenin-chemiluminescence. Right panel: The effects of difference enzyme inhibitors on the levels of ROS production by aging human brain (100%). n = 6-8 individual/group. *P<0.05 for indicated values versus young values. †p<0.05 for indicated values versus the values without inhibitor in the same age group.

Supplemental Figure V. Levels of lipid peroxidation in human mid-brain tissues.

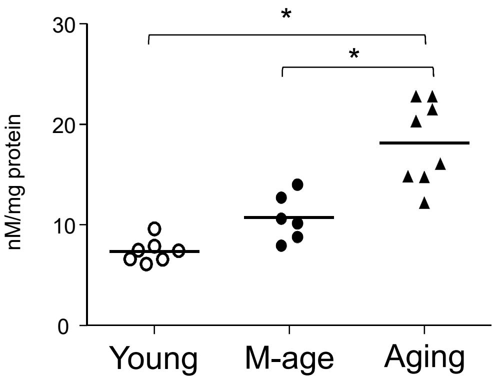

Figure V: Lipid peroxidation was detected by MDA assay using homogenate of human mid-brain tissues according to manufacturer's instruction (Sigma-Aldrich, UK). n = 6-8 individual/group. *P<0.05.

Supplemental Figure VI: Real-time PCR detection of Human Nox2 transgene only expressed in endothelial cells isolated from HuNox2Tg mice.

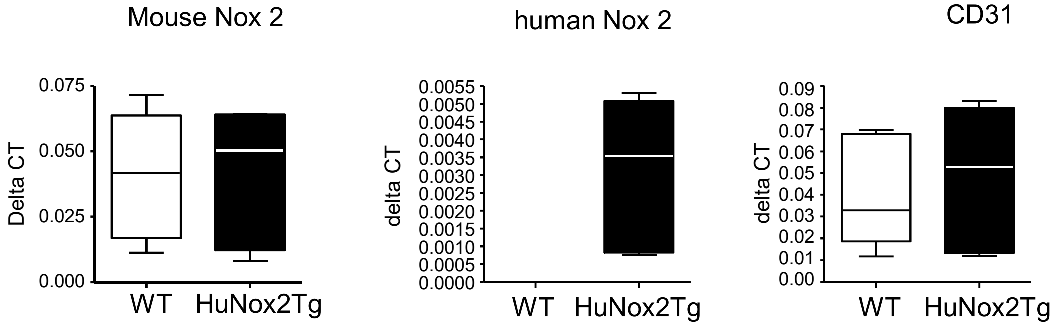

Figure VI: RNA were extracted from primary endothelial cells isolated from lungs of WT and HuNox2Tg mice and reverse transcribed into cDNA. Levels of gene expression of mouse Nox2, human Nox2, CD31 (endothelial cell marker) were detected by real time quantitative RT-PCR. Fold change was expressed as ΔC_t relative to GAPDH. n=6 isolations per group.

Supplemental Figure VII: Endothelial dependent ROS production by WT and HuNox2Tg aortas

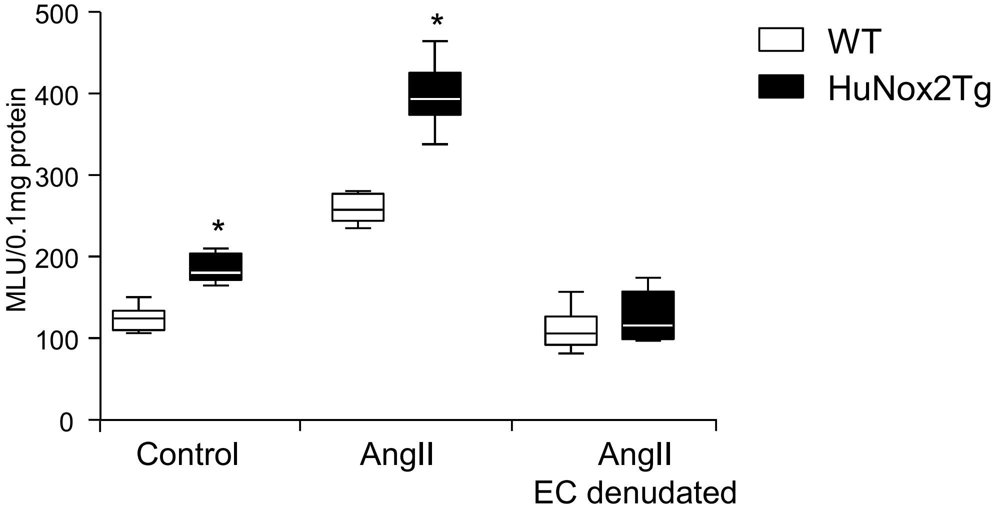

Figure VII: Aorta segments were incubated with AngII (200 µmol/L) for 4h. ROS production by aorta homogenates were detected by lucigenin-chemiluminescence. n=6 per group. *P<0.05 for indicated values versus WT values in the same treatment group.

Supplemental Figure VIII: *In situ* hybridation detection of endothelial expression of human Nox2 mRNA in brain sections of WT and HuNox2Tg mice by RNAscope.

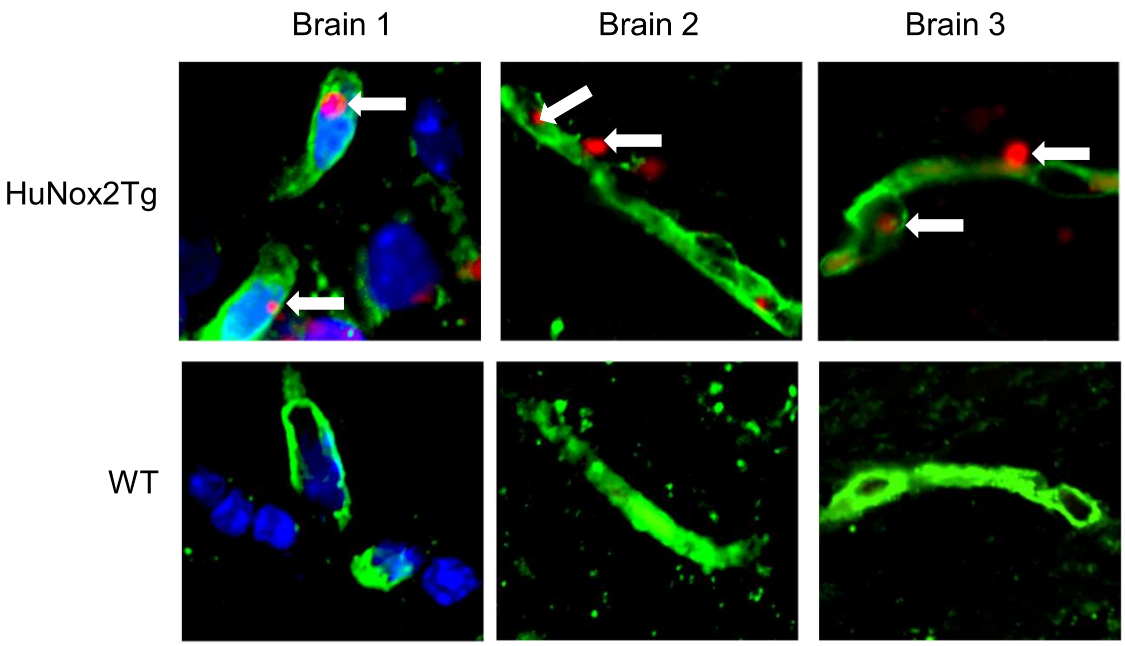

Figure VIII: RNAscope of human Nox2 mRNA was performed according to manufacturer's instruction (Advanced Cell Diagnostics Inc, USA). Cerebral endothelial cells were labelled by LE-lectin (FITC, green). The nuclei were labelled by DAPI (blue, left panel). The human Nox2 mRNA (labelled by fast red, as indicated by the arrows) were detected only in the nuclei of endothelial cells in the HuNox2Tg brain sections but not in WT brain sections.

Supplemental Figure IX: Levels of lipid peroxidation in WT and HuNox2Tg brain tissues detected by MDA assay

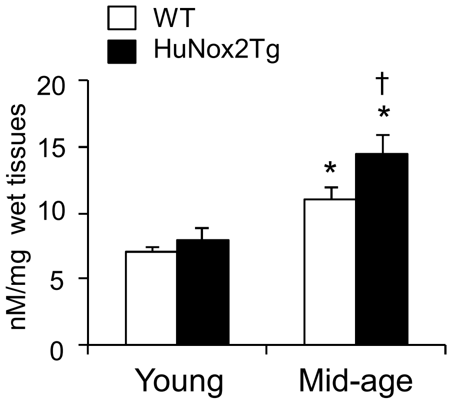

Figure IX: Homogenates of mid-brain tissues were used for MDA assay according to manufacturer's instruction (Sigma-Aldrich, UK). n=6 per group. *P<0.05 for indicated values versus young values in the same genetic group. †p<0.05 for indicated values versus WT values in the same age group.

Supplemental Table 1: List of antibodies used in the study

**Antibodies**	**Host & Type**	**Dilution**	**Company**	**Catalogue No.**
CD31	Goat polyclonal	1:500 (IF)	Santa Cruz	sc-1506
NeuN	Mouse polyclonal	1:500 (IF)	Millipore	MAB377
α-tubulin	Mouse monoclonal	1:1000	Santa Cruz	sc-53646
Nox1	Goat polyclonal	1:1000	Santa Cruz	sc-5821
Nox2	Rb polyclonal	1:1000	Santa Cruz	sc-20782
Nox4	Rb polyclonal	1:1000	Santa Cruz	sc-30141
p22phox	Goat polyclonal	1:1000	Santa Cruz	sc-11712
p40phox	Mouse monoclonal	1:1000	Santa Cruz	sc-48376
p47phox	Rb polyclonal	1:1000	Santa Cruz	sc-14015
Phos-p47phox (Ser359)	Rb polyclonal	1:500	Sigma	SAB4504289
p53	Mouse monoclonal	1:500	Santa Cruz	sc-126
p67phox	Goat polyclonal	1:1000	Santa Cruz	sc-7663
Rac-1	Rb polyclonal	1:1000	Santa Cruz	sc-217
Total-p38MAPK	Rb polyclonal	1:1000	Santa Cruz	sc-535
phos-p38 MAPK (Thr180/Tyr182)	Rb polyclonal	1:500	Cell Signalling	9211
Total-ERK1/2	Rb polyclonal	1:1000	Santa Cruz	sc-292838
Phos-ERK1/2 (Thr202/Tyr204)	Rb polyclonal	1:500	Sigma	SAB4301578
H2AX	Rb polyclonal	1:1000	Cell Signalling	2595
Phos-H2AX (Ser139, γH2AX)	Rb polyclonal	1:500	Cell Signalling	2577
Total-JNK	Mouse monoclonal	1:500	Santa Cruz	sc-7345
Phos-JNK (Thr 183/Tyr 185)	Goat polyclonal	1:500	Santa Cruz	sc-12882
p-MARCKS (Ser152/156)	Rb polyclonal	1:1000	Sigma-Aldrich	07-1238
Anti-goat IgG		1:2000	Sigma	098K4770
Anti-mouse IgG		1:2000	Seracare-KPL	474-1806
Anti-rabbit IgG		1:2000	Seracare-KPL	5450-0010

Supplemental Table 2. Demographics of human brain tissues

ID	Age	Sex	Ethnic	Cause of Death	Comorbidity	PMI (h)
Young n=7						
SD008/12	25	male	Caucasian	Accident	Schizophrenia	53
SD042/12	29	male	Caucasian	Accident	Nil known	44
SD009/05	34	male	Caucasian	Drug overdose	Depression	79
SD026/16	37	female	Caucasian	Myocardial fibrosis	Nil known	126
SD022/16	39	male	Caucasian	IHD	Nil known	86
SD038/16	39	male	Caucasian	Accident	Depression	76
SD004/12	39	male	Caucasian	Accident	Nil known	61
Mid-age n=6						
SD044/12	46	male	Caucasian	IHD	Hypertension	52
SD023/12	50	male	Caucasian	IHD	High cholesterol	45
SD038/12	51	male	Caucasian	Cardiac aneurysm	MI	45
SD037/12	52	male	Caucasian	Traffic accident	Nil known	91
SD035/12	54	male	Caucasian	MI	Hypertension, MI	74
SD020/12	56	male	Caucasian	IHD	Hypertension	44
Aging n=8						
SD049/12	60	male	Caucasian	IHD	Diabetes Hypertension	54
SD005/14	63	male	Caucasian	Lung cancer	Smoker	42
SD034/15	69	male	Caucasian	IHD	Hypertension High cholesterol	49
SD012/17	71	female	Caucasian	Accident	Depression, schizophrenia	95
SD008/17	71	female	Caucasian	IHD	Hypertension Diabetes	96
SD005/17	84	male	Caucasian	Sepsis	Diabetes	23
SD036/12	75	male	Caucasian	IHD	Nil known	78
SD055/12	76	male	Caucasian	MI	Hypertension	90

PMI=post-mortem interval; IHD=Ischemic heart diseases; MI=myocardial infarction
